# On-Chip Single-Cell Bioelectrical Analysis for Identification of Cell Electrical Phenotyping in Response to Sequential Electric Signal Modulation

**DOI:** 10.3390/bios12111037

**Published:** 2022-11-17

**Authors:** Seungyeop Choi, Insu Park, Sang Hyun Lee, Kang In Yeo, Gyeongjun Min, Sung-Hun Woo, Yoon Suk Kim, Sei Young Lee, Sang Woo Lee

**Affiliations:** 1 Department of Biomedical Engineering, Yonsei University, Wonju 26493, Republic of Korea; 2 Holonyak Micro and Nanotechnology Laboratory, University of Illinois at Urbana-Champaign, Urbana, IL 61801, USA; 3 Department of Biomedical Engineering, Konyang University, Daejeon 35365, Republic of Korea; 4 Department of Biomedical Laboratory Science, Yonsei University, Wonju 26493, Republic of Korea

**Keywords:** dielectrophoresis, DEP crossover frequency, membrane breakdown, electrical phenotype, electroporation

## Abstract

In recent years, an interesting biomarker called membrane breakdown voltage has been examined using artificial planar lipid bilayers. Even though they have great potential to identify cell electrical phenotyping for distinguishing similar cell lines or cells under different physiological conditions, the biomarker has not been evaluated in the context of living cell electrical phenotyping. Herein, we present a single-cell analysis platform to continuously measure the electric response in a large number of cells in parallel using electric frequency and voltage variables. Using this platform, we measured the direction of cell displacement and transparent cell image alteration as electric polarization of the cell responds to signal modulation, extracting the dielectrophoretic crossover frequency and membrane breakdown voltage for each cell, and utilizing the measurement results in the same spatiotemporal environment. We developed paired parameters using the dielectrophoretic crossover frequency and membrane breakdown voltage for each cell and evaluated the paired parameter efficiency concerning the identification of two different breast cancer cells and cell drug response. Moreover, we showed that the platform was able to identify cell electrical phenotyping, which was generated by subtle changes in cholesterol depletion-induced cell membrane integrity disruption when the paired parameter was used. Our platform introduced in this paper is extremely useful for facilitating more accurate and efficient evaluation of cell electrical phenotyping in a variety of applications, such as cell biology and drug discovery.

## 1. Introduction

The dielectric properties of a living cell membrane inside a microfluidic device are indicators of their physiological status, which is closely connected with cell state and function. Several previous studies have shown that changes in these dielectric properties are linked to processes such as ion channel activation [1,2]; membrane fusion; and budding and flip-flop [3,4], which contribute to the characterization of cell circadian rhythm [1,5]; cell progression [6,7]; cell viability [8,9,10]; and cell malignancy [11,12]. Recent advances in microfluidic and micro/nanotechnology have enabled the detection of cell membrane dielectric response at a whole-cell level, which makes it an attractive label-free biomarker candidate for discriminating cell populations for stem cell differentiation [13,14], leukocyte activation [15], and circulating tumor cell existence [16]. Consequently, technologies for measuring the dielectric properties of the cell membrane are in great demand.

Techniques including impedance cytometry, electrorotation, and dielectrophoresis are used to measure the cell membrane dielectric response through phenomenological parameters, such as impedance amplitude and phase, electrorotation torque, and dielectrophoretic force, respectively [17]. These techniques evaluate an interfacial polarization of which the degree is determined depending on an AC signal applied to the cell membrane that allows intrinsic membrane dielectric properties to be extracted. The membrane dielectric properties examined with these techniques can be used as electrical phenotyping to identify cell geometrical and/or physiological alterations (e.g., cell volume, membrane integrity, constitutive elements of the membrane, and ion channel activation [1,18,19,20,21,22,23]) in response to environmental stresses.

Of these electrical phenotyping techniques, dielectrophoresis (DEP) is attractive since it enables the simultaneous observation of numerous cells in the same experimental conditions and much less fabrication complexity to build electrode structures inside a microfluidic device [24]. Hence, many studies have used DEP techniques for the identification of cell electrical phenotyping through the examination of dielectric properties [25,26,27,28]. However, when DEP techniques are used for electrical phenotyping, most studies have focused on the electric frequency-dependent cell response even though the membrane dielectric properties for electrical phenotyping are affected not only by the response of dielectric polarization, but also by the accumulation of electrical charge on the membrane that is manipulated by the frequency and amplitude of the applied AC signal [29,30,31]. 

In recent years, an interesting marker has been introduced that can be applied to examine membrane dielectric properties using the electrical charge accumulation of a cellular membrane manipulated by the amplitude of an applied AC signal. When the amount of electrical charge on the lipid bilayer membrane accumulated by the applied AC signal exceeds an acceptable transmembrane potential (i.e., hyperpolarization), the membrane ruptures [32]. While the amplitude of the applied AC signal is increased beyond critical hyperpolarization, the specific amplitude generating the membrane rupture is called membrane breakdown voltage (*V*_mbd_). *V*_mbd_ varies with membrane dielectric properties, such as membrane integrity and quality, including the membrane effect of physical stimulation (e.g., accumulated electrical charges) and constituents (e.g., dipalmitoyl phosphatidylcholine and cholesterol) [33,34,35]. Therefore, *V*_mbd_ is a promising and attractive marker for identifying cellular electrical phenotyping. However, to our knowledge, *V*_mbd_ has not been used as a marker for identifying living cell electrical phenotyping.

In this paper, we present a dielectrophoretic (DEP) micro-chip platform to sequentially measure the crossover frequency (*f*_co_) and *V*_mbd_ of living cells, which are related to dielectric properties, and identify cell electrical phenotype according to linear AC frequency and voltage modulation under the same experimental conditions in facile and robust ways. To evaluate the performance of the platform on the identification of cellular electrical phenotyping, we applied *f*_co_, *V*_mbd_, or a paired parameter of *f*_co_ and *V*_mbd_, to distinguish MDA-MB-231 and SK-BR-3 breast cell lines. In addition, the distinguishable sensitivity of the cholesterol-depleted methyl-β-cyclodextrin (MβCD) drug response of the MCF-7 cell line was evaluated using the three parameters. Interestingly, the differentiation between the two breast cancer cell lines and the classification of the MβCD drug response for MCF-7 cells was more effective when the developed parameter using *f*_co_ and *V_mbd_* was employed. Overall, our developed platform can be used as a powerful tool to quantify cell type and membrane alteration by drug response in an easy, robust, and accurate way.

## 2. Materials and Methods

### 2.1. Materials and Reagents

Silicon dioxide wafers were bought from i-Nexus, Inc. (Seongnam, Republic of Korea). Polydimethylsiloxane (PDMS) was prepared using the commercial sylgard 184 elastomer and its curing agents from Dow Corning (Midland, MI, USA). Microscope cover-glass was purchased from Marienfeld (Lauda-Königshofen, Germany). Trypsin-EDTA solution (0.25%, Cat. No. 25200-072) was bought from Gibco; Thermo Fisher Scientific Inc. (Waltham, MA, USA). Cell culture medium was prepared using Dulbecco’s modified Eagle’s medium (DMEM, Cat. No. 11995-065, Gibco) supplemented with fetal bovine serum (FBS, Cat. No. 16000-044, Gibco) and penicillin-streptomycin (Cat. No. 15140-122, Gibco). Methyl-β-cyclodextrin (MβCD, Cat. No. C4555-1G) powder was bought from Sigma-Aldrich; Merck KGaA (Darmstadt, Germany). DEP buffer solution was prepared using deionized water (water purification system, Sartorius AG, Gottingen, Germany) supplemented with sucrose powder (8.6% *w*/*w*; Cat. No. SB0498, Bio Basic Inc., Markham, ON, Canada), D-Glucose powder (0.3% *w*/*w*; Cat. No. GB0219, Bio Basic Inc.), bovine serum albumin powder (BSA; 1.0 mg/mL, Bovogen Biologicals, Essendon, VIC, Australia), and phosphate-buffered saline solution (PBS; 0.2% *v*/*v*, Cat. No. 20012-027, Gibco).

### 2.2. Chip Preparation

The chip electrode used was designed to generate a partial divergence array of the square gradient of the electric field vector, which can isolate randomly distributed cells by dielectrophoretic (DEP) force. This chip was fabricated through sequential photolithography, chromium lift-off, and wet etching (see Supplementary Materials Method S1 and our previous reports [20] for further detail). Before the dielectrophoresis experiment, we cleaned the chip using solvent solution (20 min in each acetone and methanol solution), piranha solution (25 min in a solution of sulfuric acid and hydrogen peroxide (H_2_SO_4_:H_2_O_2_ = 3:1)), and deionized water. Subsequently, this chip was combined with the donut-shaped polydimethylsiloxane (PDMS) reservoir with a 10 mm-external diameter and a 5 mm-internal diameter hole cut out of a PDMS film. When the DEP experiment was repeated, we repeated the clearing process and combined new donut-shaped PDMS reservoirs with the chip. 

### 2.3. Cell Preparation

Each human breast cancer cell line, including SKBR-3, MDA-MB-231, and MCF-7 cells, was incubated in the fresh cell culture medium (see Materials and Reagents) at 37 °C in a humidified atmosphere with 5% CO_2_. Before the DEP experiment, cells were seeded on a six-well cell culture plate at a concentration of 2 × 10^5^ cells/mL and incubated for 24 h in the cell incubator (Thermo Fisher Scientific Inc.). The cell culture dishes were then washed with fresh cell culture medium, harvested using the Trypsin/EDTA solution, and exchanged into DEP buffer solution (see Materials and Reagents) by centrifugation.

### 2.4. Drug Treatment

We dissolved 0.1 g of MβCD powder into 2 mL of PBS, resulting in a 37.8 mM PBS solution. Sequentially, the solution was diluted with 2.5 mM, 5 mM, or 10 mM MβCD in serum-free DMEM solution. MCF-7 cells were treated with each MβCD solution for 2 h and removed using fresh DMEM solution. The treated cells were then harvested using Trypsin/EDTA solution and exchanged into DEP buffer solution (see Materials and Regents) by centrifugation.

### 2.5. Experimental Setup

The PDMS reservoir-connected electrode chip was injected with a 15 µL-DEP buffer solution containing cells. The reservoir was then enclosed using a cover glass to observe the cell motion without the solution evaporating. It was placed on a plate on the custom probe station (Modusystems Inc., Hanam, Republic of Korea) and fixed strongly with a vacuum pump. Once the cells had settled onto the chip surface, the AC input signal (AC 2 V_p-p_, 1 kHz sine wave) was applied to separate the cells by negative DEP force, which was assessed via a bright microscope-connected charge-coupled device (CCD; Motionscope M3, Redlake, San Diego, CA, USA). Finally, the cell motion was recorded where the input signal sequence (see next section for more detail) was applied in the LabVIEW (National Instruments, Austin, TX, USA)-based automated system, which synchronized electric signal transmission and microscope image recording.

### 2.6. Electric signal Configuration

We designed the electric signal configuration to obtain a continuous single-cell measurement of DEP crossover frequency (*f*_co_) by input frequency shift and membrane breakdown voltage (*V*_mbd_) by input voltage shift in the same environment. In detail, the input signal for the measurement of *f*_co_ was controlled as follows: (1) AC 1 kHz and 2 V_p-p_ were applied to align cells by negative DEP force; (2) with AC 2 V_p-p_, the AC frequency was linearly increased from 1 kHz to 41 kHz at 100 Hz/s. Subsequently, the input signal was controlled as follows for the measurement of *V*_mbd_: (1) AC 41 kHz and 2 V_p-p_ were applied to align cells by positive DEP force; (2) with AC 41 kHz, the AC voltage was linearly increased from 2 V_p-p_ to 12 V_p-p_ at 0.05 V_p-p_/s. The measured *f*_co_ and *V*_mbd_ were obtained through image analysis of the cell motion recording considering the input signal sequence (see next sections for more detail).

### 2.7. Determination of DEP Crossover Frequency (f_co_)

Using cell image analysis, the experimental DEP *f*_co_ of cells was defined as the initial frequency when a cell mobility alteration is induced by the transition of DEP force direction, as described in previous reports [20,36,37]. Briefly, on an electrode, cells’ movements were labeled and image sequences of the movements were tracked via the custom-optimized image segmentation algorithm. Subsequently, our cells of interest, which aligned with the divergence region of negative DEP force and then escaped by the transition of DEP force direction, were automatically selected by the comparison between the region into which the cell moved and the user-defined electrode region. At each cell image of interest, the average pixel intensity (i.e., brightness) within the region was collected in ascending frame order. The average brightness of all frames was then interrogated by evaluating the transition frame where the intensity was greater than three times the standard deviations of the cell image intensity distribution. Finally, the determined frame number of each cell was converted to its corresponding frequency number. This procedure has been described in more detail in our previous reports [20,36].

### 2.8. Determination of Membrane Breakdown Voltage (V_mbd_)

We derived a membrane breakdown voltage parameter using image correlation analysis conducted through MATLAB (MATLAB 2021b, Math Works GK, MA, USA). We focused on the continuous measurement of DEP *f*_co_ and *V*_mbd_ for individual cells in the same environment. As such, cells of interest were limited to those for which we succeeded in acquiring a DEP *f*_co_ value in advance. We selected one reference image per cell in which its image showed an intact cell outline before the initiation of membrane breakdown by increasing input voltage. Subsequently, the two-dimensional correlation coefficient (*γ*) was calculated between this reference image and another cell image sequence during voltage modulation (i.e., 2 V_p-p_ to 12 V_p-p_) as follows:

(1) $$\gamma =\frac{\sum_{m}\sum_{n}\left(R_{mn}-\bar{R}\right)\left(T_{mn}-\bar{T}\right)}{\sqrt{(\sum_{m}\sum_{n}{\left(R_{mn}-\bar{R}\right)}^{2})(\sum_{m}\sum_{n}{\left(T_{mn}-\bar{T}\right)}^{2}})}$$

where *R* and *T* are the reference image at 2 V_p-p_ and the test image concerning from 2 V_p-p_ to 12 V_p-p_, respectively. The subscript ‘*m*’ and ‘*n*’ represents the size of the column and row of the image matrix.

In this coefficient calculation, when the calculated correlation coefficient value is 1, we can evaluate that the pixel intensity distribution within a test image is identical to that of the reference image. When the calculated value is smaller or greater than 1, we can evaluate that the test image is more different from that of the reference image. These coefficients, spanning all examined cells, were calculated to obtain the absolute coefficient value following “|1-γ|” and collected in ascending frame order. The absolute coefficient was then interrogated by evaluating the transition frame where a coefficient was greater than three times the noise standard deviations within the defined coefficient interval where the absolute coefficient stayed near zero. Finally, the determined frame number of each cell was converted to its corresponding voltage number (i.e., *V*_mbd_). This procedure is described in more detail in Supplementary Materials Method S2.

### 2.9. Statistical Analysis

All experimental data were analyzed using Origin Pro software (Origin Lab, Northampton, MA, USA). MATLAB software was utilized for the determination and calculation of overlapping regions between two intersecting probability distributions.

## 3. Results and Discussion

### 3.1. Platform Concept and Working Design

**Figure 1:**
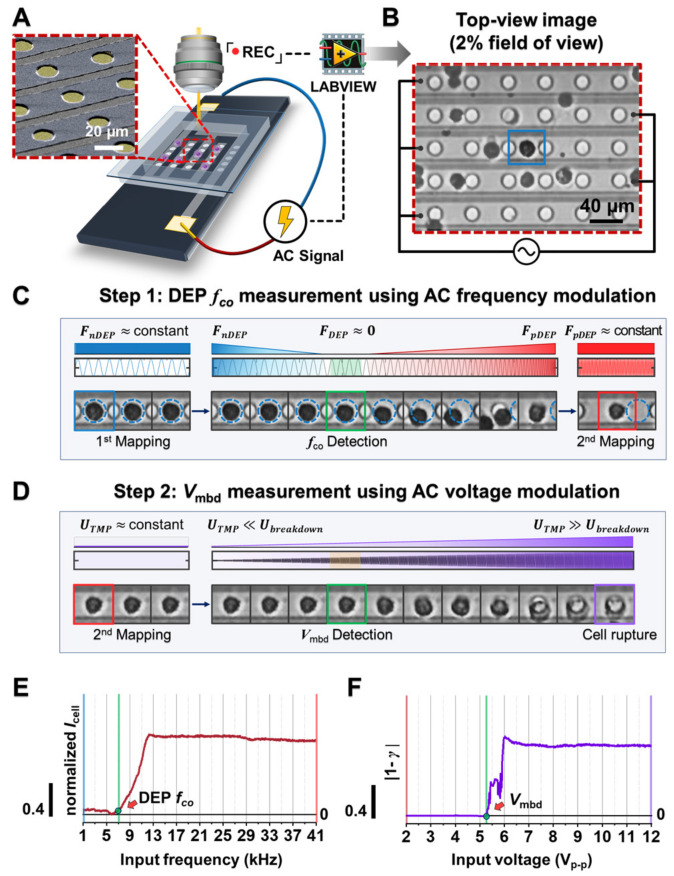
Design and working principle of the proposed platform. (**A**) Schematic illustration of the platform. The red inset is the SEM image of the chip substrate (the original SEM image was also attached in Figure S1 in Supplementary Materials). (**B**) The representative top-view image demonstrating that cells were grounded and trapped between the neighboring circular electrodes under the influence of negative and positive DEP forces. Please also see Figure S4 in Supplementary Materials for a detailed description of the cell trapped position. (**C**,**D**) Signal application mode for monitoring cell dielectric behavior. Using cell tracking analysis, each cell was labeled with DEP crossover frequency and membrane breakdown voltage, which was induced as a phenomenological parameter from the sequential electric signal modulation of AC frequency (**C**) and AC voltage (**D**). (**E**,**F**) Dielectric response of a cell during the increase of AC frequency and AC voltage, respectively. In each signal, the transition moment (green line) was determined as DEP *f*_co_ (**E**) and *V*_mbd_ (**F**) (see Materials and Methods).

The schematic illustration of a dielectrophoresis (DEP) trapping-based integrated platform is shown in Figure 1A. Such a platform consists of a micro interdigitated electrode array chip with circular shapes (a more detailed structure is shown in Figure S1), an AC signal generator, and a microscope connected to a CCD camera. An example of random cells (e.g., black circles) on a chip individually separated and trapped between the neighboring circular electrodes (e.g., white circles) by DEP force [20,21] is shown in Figure 1B, where the AC electric signal, 1 kHz and 2 V_p-p_, was applied under a custom-built LABVIEW control. Figure 1C,D show the observed single-cell image sequence varying with AC frequency and voltage modulation, respectively. First, to acquire more consistent tendencies of cell behavior according to the electric signals, we confined the cells of interest that were initially trapped the midpoint between the neighboring circular electrodes, where is the local converged minimized region of the electric field gradient direction using negative DEP force [20,36,37,38,39]. Next, through AC frequency modulation, the trapped cells escaped and were then trapped within a circular electrode that is the local converged maximized region of the electric field gradient direction by positive DEP force (Figure 1C and Figure S4) [20,36,37,38,39]. Sequentially, the amplitude of the applied AC signal was increased. As a result, the shapes of such trapped cells were blurred due to cell membrane rupture (Figure 1D).

To evaluate the cell dielectric response to each electric signal modulation, we determined the DEP crossover frequency (*f*_co_) and membrane breakdown voltage (*V*_mbd_) from an analysis of the image brightness variation within the region of interest where cells were trapped by negative or positive DEP force (Figure 1E,F). Briefly, while input frequency was incremented, the region of interest was the negative DEP force-induced cell region (blue dotted circle in Figure 1E). We compared the brightness intensity of this region and could determine *f*_co_ when the intensity sharply increased for the first time (see Materials and Methods). After a cell was trapped on the circular electrode, it was imaged at 2 V_p-p_ (red box in Figure 1D). We set this image as the reference and calculated the 2-D correlation coefficient through the comparison between the reference image and others, leading to the determination of *V*_mbd_ (see Materials and methods), indicated by the blue dotted circle in Figure 1F.

### 3.2. Different Breast Cancer Cell Lines Were Distinguished Using the Developed Platform

**Figure 2:**
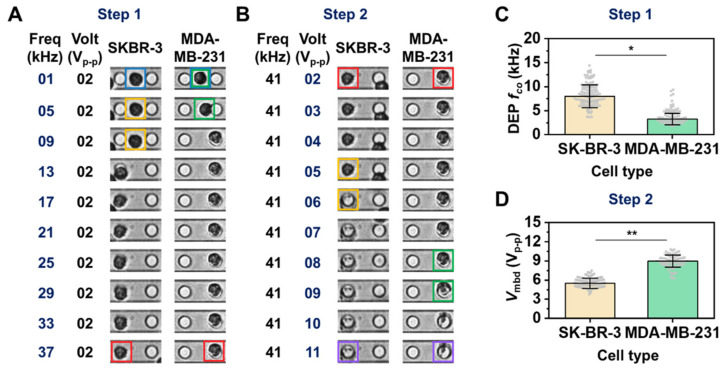
Comparison of *f*_co_ and *V*_mbd_ for SKBR-3 and MDA-MB-231 cells. (**A**,**B**) Time sequential images showing cell transitions and alterations over increments of input frequency (**A**) and input voltage (**B**). The blue color boxes show two representative cell that trapped under negative DEP force. The yellow and green color boxes in (**A**) mark the places where SKBR-3 cell and MDA-MB-231 cell were departed from the center of blue color box position by the transition of DEP force direction, respectively. The yellow and green color boxes in (**B**) mark the places where the transition of brightness within region of SKBR-3 cell and MDA-MB-231 cell was observed by cell membrane rupture following the increase of the input voltage, respectively. The red color boxes show two representative cell that trapped under positive DEP force. The purple color boxes show both ruptured cells by membrane breakdown. (**C**,**D**) DEP *f*_co_ (**C**) and *V*_mbd_ (**D**) for the comparison between the above two cell lines. Each parameter was obtained using cell image analysis (see Materials and Methods). Each gray dot denotes DEP *f*_co_ and *V*_mbd_ data for an individual cell. The two sample *t*-test (two-tailed) was used to check the significance of a difference (* *p* and ** *p* < 0.001). The measurement procedure to obtain the average *f*_co_ and *V*_mbd_ with standard division was repeated three times. More detailed measurement information is provided in Supplementary Materials Tables S4 and S5.

We next investigated whether such parameters, *f*_co_ and *V*_mbd_, could distinguish two different cells by comparing their membrane dielectric properties. For the investigation, we prepared two different breast cancer cell lines, SK-BR-3 cells (weakly invasive breast cancer cells) and MDA-MB-231 cells (highly invasive breast cancer cells), with similar size distributions (Figure S3) but different dielectric properties. The dielectric responses of such different cells were sequentially recorded while the input frequency and voltage sequentially increased. Figure 2A represents the typical cell movements with respect to increases in input frequency and shows that SKBR-3 cells escaped from the negative DEP trap more slowly than did MDA-MB-231 cells (see yellow and green boxes, respectively, in Figure 2A). Moreover, concerning AC voltage, the membrane outline of SKBR-3 cells disappeared more quickly compared to that of MDA-MB-231 cells (see yellow and green boxes, respectively, in Figure 2B) alongside the applied AC voltage. Thus, using both AC frequency and voltage modulation, it is possible to discriminate populations of SKBR-3 and MDA-MB-231 cells. To investigate this tendency quantitatively on our platform, we characterized the *f*_co_ and *V*_mbd_ of SKBR-3 cells and MDA-MB-231 cells, as shown in Figure 2C,D. The average (±standard deviation) of *f*_co_ for MDA-MB-231 cells was 3.86 ± 1.22 kHz, which is well matched to the previous reports (Figure S5) [20,40,41,42]. The average of *f*_co_ for SKBR-3 cells was also 8.24 ± 2.40 kHz (Figure 2C). Moreover, the average (± standard deviation) of *V*_mbd_ for SKBR-3 cells and MDA-MB-231 cells was 5.56 ± 0.81 V_p-p_ and 8.84 ± 0.93 V_p-p_, respectively. Both SKBR-3 cell *f*_co_ and *V*_mbd_ distributions were significantly different than those of the MDA-MB-231 cells (*, ** *p* < 0.001 in the two-sample *t*-test).

**Figure 3:**
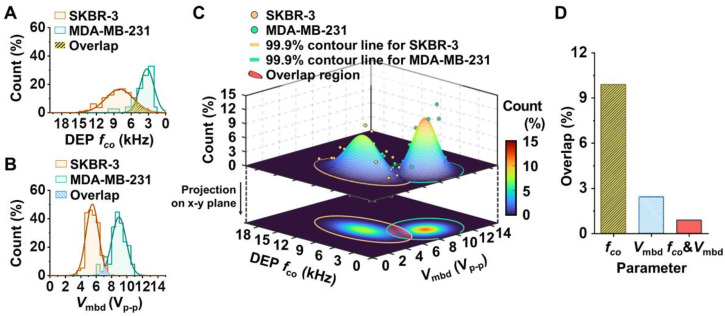
Electrical phenotyping of SKBR-3 cells and MDA-MB-231 cells. (**A**,**B**) Representative 1D histogram and Gaussian normal distribution concerning DEP *f*_co_ (**A**) and *V*_mbd_ (**B**). (**C**) Representative 2D Gaussian normal distribution of *f*_co_ and *V*_mbd_. Each dot denotes the bin frequency (%) of DEP *f*_co_ and *V*_mbd_ in each cell population. This distribution is projected on DEP *f*_co_ and *V*_mbd_ and expresses the density plot of the 2D Gaussian distribution. The yellow and green lines represent the contour line that occupies 99.9% of the count region of 2D Gaussian distribution for SKBR-3 (*n* = 113 cells) and MDA-MB-231 (*n* = 76 cells) cell populations, respectively. The red region represents the overlapping area between the two distributions. (**D**) The overlapping occupancy (%) between the two breast cancer cell population distributions using DEP *f*_co_, *V*_mbd_, and both *f*_co_ and *V*_mbd_.

However, even though the average *f*_co_ and *V*_mbd_ were clearly distinguished between the cell types, portions of the *f*_co_ and *V*_mbd_ distributions for the individual cells overlapped. The normalized Gaussian distributions (R^2^ > 0.90) that were fitted to a *f*_co_ and *V*_mbd_ histogram bin using the measured data shown in Figure 2C,D are shown in Figure 3A,B, demonstrating the intersecting region between the two distributions (e.g., the yellow-shaded and blue-shaded regions in Figure 3A,B, respectively). Upon additional quantitative analysis, the normal *f*_co_ distributions of SKBR-3 and MDA-MB-231 cells overlapped by 10% (Figure 3A). The normal *V*_mbd_ distributions of SKBR-3 and MDA-MB-231 cells overlapped by only 2% (Figure 3B), which indicates that *V*_mbd_ is a better parameter to distinguish the two cell types. Next, we applied the two-dimensional (2D) Gaussian normal distribution of *f*_co_ and *V*_mbd_ to characterize the electric phenotypic profiles of the two cell populations, as shown in Figure 3C. As a result, the overlapping density was 0.8%. The summary of the overlapping regions analyzed by the three parameters is shown in Figure 3D. Based on the results in Figure 3, the 2D paired analysis of *f*_co_ and *V*_mbd_, which are measured under the exact same environment using the developed on-chip platform, can enhance the discriminated capability of electric phenotypic profiles rather than using *f*_co_ or *V*_mbd_ alone to evaluate a mixture of cell populations.

### 3.3. Drug-Treated Breast Cancer Cells Were Distinguished Using the Developed Platform

**Figure 4:**
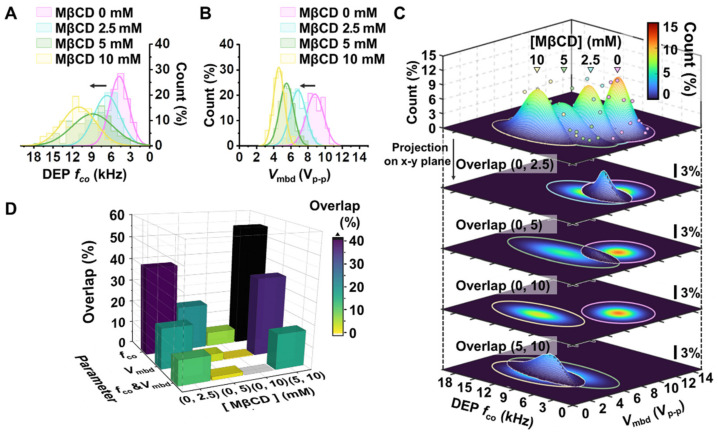
Electrical phenotyping of MCF-7 cells in response to MβCD treatment. (**A**,**B**) Representative histogram and Gaussian normal distribution concerning DEP *f*_co_ parameter (**A**) and *V*_mbd_ (**B**). Each histogram was plotted from data of 136, 129, 102, and 82 cells for 0, 2.5, 5, and 10 mM of MβCD treatment condition, respectively. (**C**) Representative 2D Gaussian distribution using both *f*_co_ and *V*_mbd_ for the MβCD-treated cell population. Each dot denotes the bin frequency (%) of DEP *f*_co_ and *V*_mbd_ in each cell population. Each color line represents the 99.9% contour line of the 2D Gaussian distribution with respect to MβCD treatment concentration. This distribution was projected onto the DEP *f*_co_-*V*_mbd_ plane and can be expressed as the density plot of the 2D Gaussian distribution. The calculated overlap count between the two distributions is represented on the projection plane. (**D**) The overlapping occupancy (%) of the population distribution between no treatment and treatment used the parameters DEP *f*_co_, *V*_mbd_, and both *f*_co_ and *V*_mbd_. The overlapping comparison of all cases is denoted in Tables S1–S3, which correspond to *f*_co_, *V*_mbd_, and both *f*_co_ and *V*_mbd_, respectively.

Methyl-β-cyclodextrin (MβCD) has been well established to induce the depletion of cholesterol molecules on the lipid membrane in adherent cell lines, such as MCF-7 breast cancer cells [20]. We treated MCF-7 breast cancer cells with MβCD to evaluate the resolution of the developed platform alongside the concentration of MβCD. In accordance with the cell tracking and cell image analysis described in the above section, we calculated and represented the Gaussian normal distributions of DEP *f*_co_ and *V*_mbd_ with respect to the concentration of MβCD treatment, as shown in Figure 4A,B. Furthermore, since the developed platform enables both DEP *f*_co_ and *V*_mbd_ parameters to be obtained at the single cell level in the exact same environment, we depicted the 2D density distribution using both parameters in easy and robust ways (Figure 4C). Next, to evaluate the preference of such parameters, *f*_co_, *V*_mbd_, and the 2D parameter *V*_mbd_ associated with *f*_co_, and distinguish drug-induced cell changes, we extracted the overlapping occupancy between cell population distributions with varying concentrations of MβCD. The comparison results are shown in Figure 4D. According to Figure 4D, the *f*_co_ could discriminate well between non-treated MCF-7 cells and drug-treated MCF-7 cells when 10 mM MβCD was introduced to the cells (e.g., 6.5% overlapped region between non-treated MCF-7 cells and 10 mM MβCD-treated MCF-7 cells), indicating that it is correct to use *f*_co_ when a large amount of drug is applied to a cell. However, the ability to distinguish between non-treated MCF-7 cells and drug-treated MCF-7 cells was dramatically reduced when the concentration of MβCD treatment decreased. The percentages of overlap at 5 mM and 2.5 mM in the comparison with non-treated MCF-7 cells were 20.3% and 39.7%, respectively. The results indicate that *f*_co_ is not a proper parameter for discrimination when using a small amount of drug. *V*_mbd_ shows better performance than *f*_co_. According to the results shown in Figure 4D, the percentage of overlap was 3.5% at 5 mM of MβCD treatment in MCF-7 cells. However, the percentage of overlap was still not clearly distinguished when 2.5 mM MβCD was introduced to MCF-7 cells (e.g., 18.2% overlapping regions between non-treated MCF-7 cells and 2.5 mM MβCD-treated MCF-7 cells). On the other hand, the overlap percentages using 2D paired analysis of jointly associated *f*_co_ and *V*_mbd_ were 0%, 1.6%, and 11.1% at 10 mM, 5 mM, and 2.5 mM, respectively. Furthermore, the 2D analysis showed good performance between drug-treated MCF-7 cells with different drug treatment concentrations between 2.5 mM-treated MCF-7 cells and 5 mM-treated MCF-7 cells or between 5 mM- and 10 mM-treated MCF-7 cells, as shown in Figure 4D. The overlapping comparison of all cases is denoted in Supplementary Materials Tables S1–S3, which correspond to *f*_co_, *V*_mbd_, and both *f*_co_ and *V*_mbd_. These results in Figure 4 indicate that the platform can distinguish subtle differences in cell electrical phenotyping when cells are deformed by the affection of a drug.

## 4. Conclusions

We developed the electric signal frequency and voltage modulation system in a dielectrophoresis trapping-based platform. The developed platform is capable of the sequential measurement of two dielectric phenomenological parameters, DEP crossover frequency (*f*_co_) and membrane breakdown voltage (*V*_mbd_), through the simultaneous manipulation of individual cells by DEP force under the same environment. Using the measurement results in the developed platform, we designed three parameters, *f*_co_, *V*_mbd_, and the combination of *f*_co_ and *V*_mbd_, and evaluated which was correct for distinguishing electric phenotype profiles of different breast cancer cell types and each cell group treated with different drug concentrations. The optimized *V*_mbd_ parameter provided a more sensitive electrical metric for discriminating such different cell groups than the conventional *f*_co_ parameter. Furthermore, the 2D paired analysis regarding the parameter combining *f*_co_ with *V*_mbd_ had the best performance for the discrimination of such phenotypic profiles, indicating that the 2D parameter could be very useful for evaluating a mixture of cell populations or detecting subtle differences in cell electrical phenotyping within a cell population. Thus, the developed platform has great promise as an efficient, label-free electrical phenotyping tool for biomedical and clinical applications requiring the characterization of cell dielectric property at the single-cell level.

## Data Availability

Not applicable.

## Supplementary Materials

The following supporting information can be downloaded at: https://www.mdpi.com/article/10.3390/bios12111037/s1, Supplementary Methods: Chip fabrication, Programmable methods for measuring *V*_mbd_, and Supplementary figures: Figure S1: The representative SEM image of the DEP electrode after chip fabrication, Figure S2: The workflow for measuring *V*_mbd_ on the DEP chip. (A) Workflow diagram of the image analysis-based *V*_mbd_ measurement. (B) The representative image guiding the cell labeling process in which each cell was assigned its name (i.e., ID), size, and center position after cell tracking analysis for the captured image sequence. (C) The example reference image list for multiple cells numbered from 574 to 683. These cell ID numbers are also shown on the middle panel image of Figure S2B. (D) Schematic overview of the determination of the membrane breakdown voltage using correlation image analysis. The representative and test sample image sequences were represented using cell cID# 625. Blue and gray color of “1-corr” value is for cID# 625, other 14 cells in Figure S2, respectively, Figure S3: The measured radius distribution of SKBR-3 and MDA-MB-231 cells, respectively. The Gaussian normal distribution is well fitted (R-square > 0.9). The statistical difference between them is not sufficient (*p* > 0.05), Figure S4: The behavior tendency of DEP-induced cells on electrode. (A). Finite element method analysis of electric field distribution with DEP force arrow at 8.8-μm height from the chip surface. (B,C) Top view image when the cells were trapped by negative and positive DEP force on our electrode, where the position between neighboring circular electrode is the local minimum region of electrical field intensity and the position within circular electrode is the local maximum region of electrical field intensity, respectively and Supplementary tables: Table S1: The evaluation of overlap count between two MβCD samples using DEP *f*_co_, Table S2: The evaluation of overlap count between two MβCD samples using *V*_mbd_, Table S3: The evaluation of overlap count between two MβCD samples using both DEP *f*_co_ and *V*_mbd_, Table S4: Biological variability in bioelectrical measurement of SK-BR-3 cells, Table S5: Biological variability in bioelectrical measurement of MDA-MB-231 cells.
