# Supplementary material for: On-Chip Single-Cell Bioelectrical Analysis for Identification of Cell Electrical Phenotyping in Response to Sequential Electric Signal Modulation
Source: Biosensors (Basel). 2022 Nov 17;12(11):1037. doi: 10.3390/bios12111037 (PMC9688586; doi:10.3390/bios12111037)
Supplement: Supplementary file 1 [file biosensors-12-01037-s001.zip › biosensors-1994701-supplementary.pdf]

Supplementary Materials Appendix for

On-chip single-cell bioelectric analysis for identification of cell electrical phenotyping in response to sequential electric signal modulation

Seungyeop Choi[1,†], Insu Park[2,3,†], Sang Hyun Lee[1,†], Sunghun Woo[4], Kang In Yeo[1], Kyeung Jun Min[1], Yoon Suk Kim[4], Sei Young Lee[1], and Sang Woo Lee[1,*]

[1] Department of Biomedical Engineering, Yonsei University, Wonju, 26493, Republic of Korea

[2] Holonyak Micro and Nanotechnology Laboratory, University of Illinois at Urbana-Champaign, Urbana, IL 61801, USA

[3] Department of Biotechnology and Bioinformatics, Korea University, Sejong, 30019, Republic of Korea

[4] Department of Biomedical Laboratory Science, Yonsei University, Wonju 26493, Republic of Korea

[†]These authors contributed equally to this work

* Corresponding author: S. W. Lee (yusuklee@yonsei.ac.kr)

This PDF file includes:

Supplementary Methods S1 to S2

Figures S1 to S4

Tables S1 to S5

Reference

1. Supplementary Methods

Method S1 | Chip fabrication

A silicon dioxide (SiO_2) 4-inch wafer was sonicated in acetone, methanol, and deionized water solution for 10 min and then dried with nitrogen gas. This wafer was soaked in piranha solution for 20 min and rinsed using deionized water. In a vacuum desiccator, hexamethyldisilazane (HDMS, Sigma Aldrich) vapor was deposited on the dried wafer for 20 min to make the wafer surface hydrophobic for photoresist (AZ GXR-601, AZ Electronics Materials, Luxembourg) adhesion. The photoresist was spin-coated onto the wafer surface at 3000 rpm for 30s and then annealed at 65 °C for 30 s. Subsequently, it was exposed to UV light through a photomask for 3 s using a mask aligner. The no-exposed photoresist was removed using AZ 300 MIF developer (AZ Electronics Materials). The chromium (Cr) electrode was deposited with 100-nm thickness on this removal region using thermal evaporation. After that, the Cr-patterned wafer was insulated by an 800-nm-thick layer of SiO2 using a plasma-enhanced chemical vapor deposition process to minimize the electrode chemical reaction. We repeated the photolithography process again to develop the array of 20-μm-sized circles and electrode pad for applying external signal. After the hard bake, the PR-deposited wafer was etched in buffered oxide etch solution, and an array of 20-μm-sized circular etched holes and the electric pad hole were made. Figure S1 shows the representative SEM image of our chip after these fabrication procedures.

Method S2 | Programmable methods for measuring V_{mbd}

To characterize the variation of cell outline, as shown in Figure 2, we considered the membrane breakdown voltage (V_{mbd}), where the change of a cell's outward appearance was initiated using the image analysis technique. Figure S2 provides the procedure of our image analysis for determining V_{mbd}. In the first step (Figure S2B), we exploited the cell trajectory to observe the variation of cell outline. From the cells of interest, using the optimized cell tracking analysis on our DEP chip [1], we excluded (1) adjoining cells and (2) untrapped cells within a circular electrode. The middle image in Figure S2B is an example result of the first step. The cell of interest is marked with a red point in its center. Cells with blue points or no labeling were automatically excluded for the reasons above. Cell name (i.e., ID#) and measurements of size and center location per frame (x, y) were saved. In the second step, we selected the reference image as the last assigned frame image before the increase of input voltage. As shown in Figure S2C, there are several reference cell images on the cropped image in Figure S2B at 2 $V_{p\text{-}p}$ 41 kHz of input. In the third step, after reference image selection, the correlation coefficient was calculated. For example, the reference image, which is cell cID# 625, is shown in the blue outlined image sequence in Figures S2C and D. The test sample image of this cell for V_{mbd} evaluation is shown in the magenta outlined image sequence in Figure S2D. The calculation results are shown in the bottom graph of Figure S2D with the specific cells in Figure S2C. This coefficient was abruptly increased with respect to the change in cell images toward the nearly transparent image. Thus, we technically define the intersection between the first gradually increasing region and the suddenly increasing region using the MATLAB library functions. Subsequently, the best linear regression model is found out within the interval from the initial voltage to 80% of intersection voltage. Finally, we calculated the standard deviation of difference between the absolute coefficient and the estimated regression value for each voltage number and determine the V_{mbd} by evaluating the transition frame where a difference value was greater than the three times of the calculated noise standard deviations.

2. Supplementary Figures

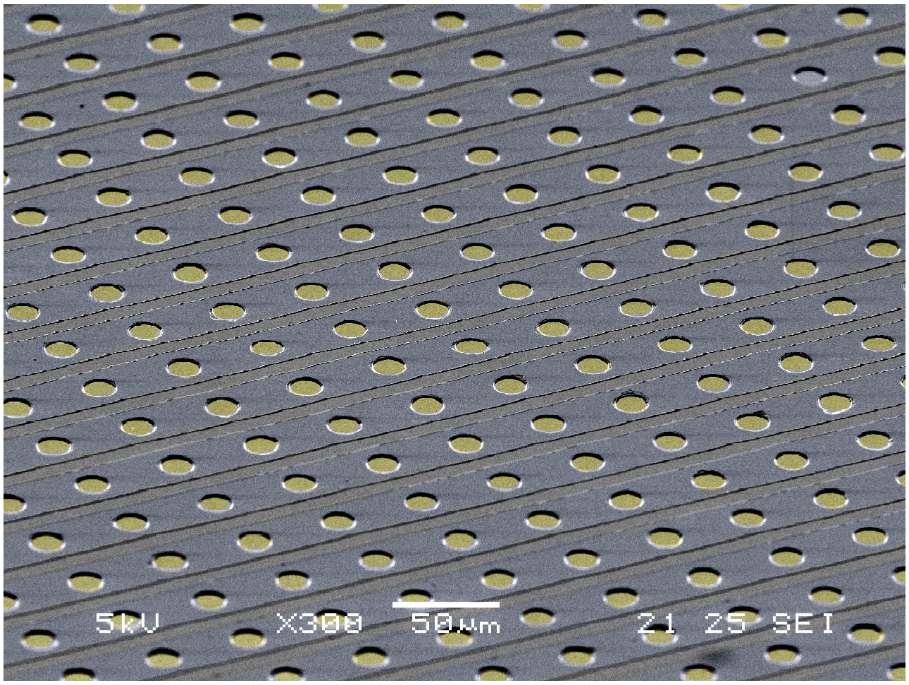

Figure S1. The representative SEM image of the DEP electrode after chip fabrication.

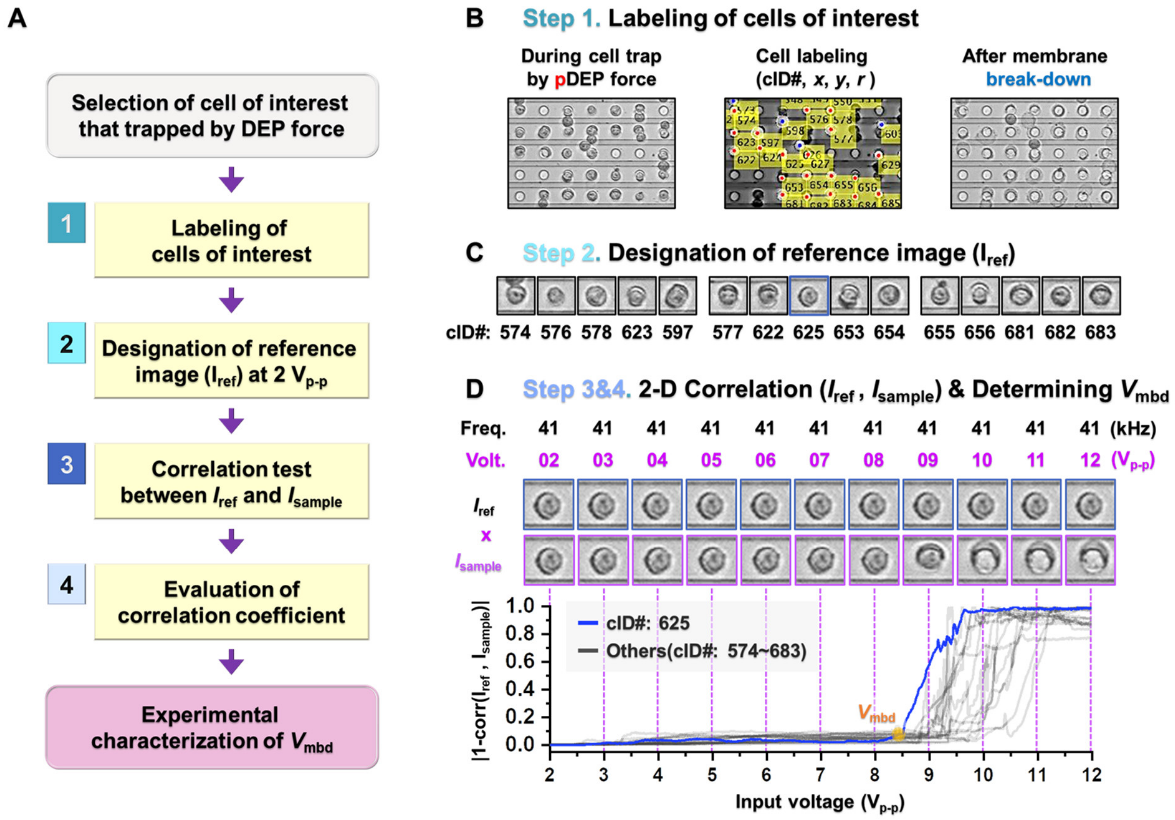

Figure S2. The workflow for measuring V_{mbd} on the DEP chip. (**A**) Workflow diagram of the image analysis-based V_{mbd} measurement. (**B**) The representative image guiding the cell labeling process in which each cell was assigned its name (i.e., ID), size, and center position after cell tracking analysis for the captured image sequence. (**C**) The example reference image list for multiple cells numbered from 574 to 683. These cell ID numbers are also shown on the middle panel image of Figure S2B. (**D**) Schematic overview of the determination of the membrane breakdown voltage using correlation image analysis. The representative and test sample image sequences were represented using cell cID# 625. Blue and gray color of "1-corr" value is for cID# 625, other 14 cells in Figure S2, respectively.

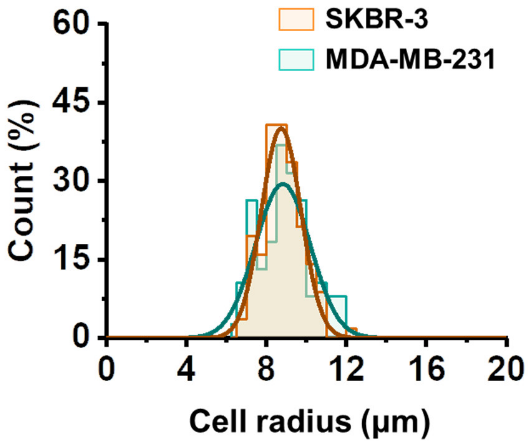

Figure S3. The measured radius distribution of SKBR-3 and MDA-MB-231 cells, respectively. The Gaussian normal distribution is well fitted (R-square > 0.9). The statistical difference between them is not sufficient (P>0.05).

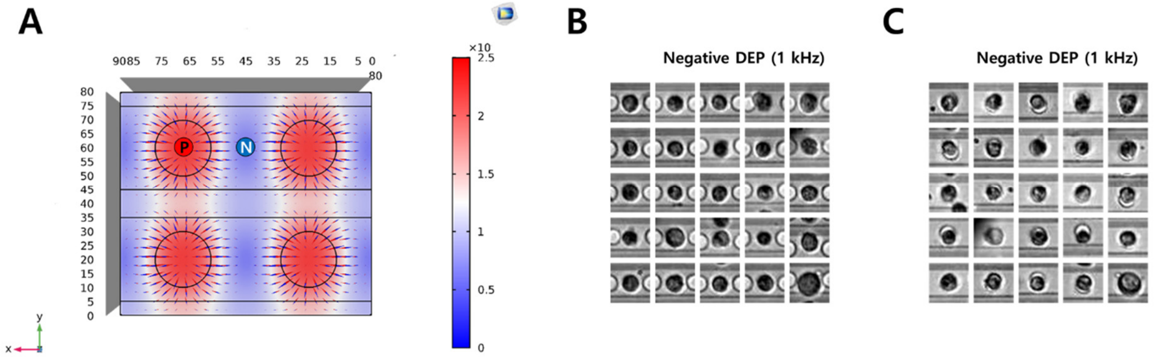

Figure S4. The behavior tendency of DEP-induced cells on electrode. (A). Finite element method analysis of electric field distribution with DEP force arrow at 8.8-μm height from the chip surface. (B, C) Top view image when the cells were trapped by negative and positive DEP force on our electrode, where the position between neighboring circular electrode is the local minimum region of electrical field intensity and the position within circular electrode is the local maximum region of electrical field intensity, respectively.

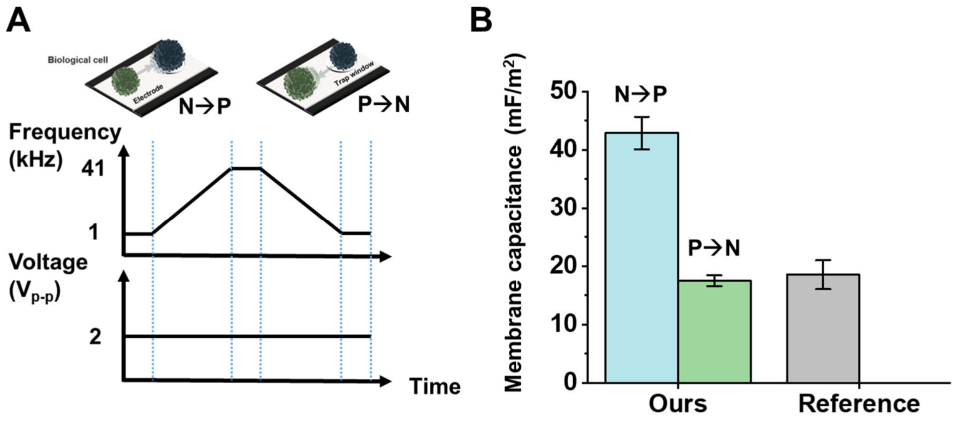

Figure S5. The comparison of membrane capacitance of MDA-MB-231 with other literature. (A) the schematic procedure of frequency modulation for DEP crossover frequency measurement on our platform. (B) the average membrane capacitance of MDA-MB-231 breast cancer cell. The membrane capacitance was induced from DEP crossover frequency that obtains in the three independent experiment (*n*=76, 105, 100 cells). The reference membrane capacitance of MDA-MB-231 was averaged from 21.2 mF/m^2, 16.3 mF/m^2, 18.1 mF/m^2 in three previous literatures [2-4]. Due to cell variable dielectric property which validated in our previous report [5], we additionally performed to measure the DEP crossover frequency of MDA-MB-231 by the decrease from 41 kHz to 1 kHz (P→N) in order to compare other previous reports.

3. Supplementary Tables

Table S1. The evaluation of overlap count between two MβCD samples using DEP f_{co} .

Only DEP f_{co}	**0 mM**	**2.5 mM**	**5 mM**	**10 mM**
**0 mM**	100%	-	-	-
**2.5 mM**	39.7%	100%		-
**5.0 mM**	20.3%	46.2%	100%	-
**10 mM**	6.5%	21.1%	55.0%	100%

Table S2. The evaluation of overlap count between two MβCD samples using $V_{mbd.}$

Only V_{mbd}	**0 mM**	**2.5 mM**	**5 mM**	**10 mM**
**0 mM**	100%	-	-	-
**2.5 mM**	18.2 %	100%	-	-
**5.0 mM**	3.5%	28.3%	100%	**-**
**10 mM**	0.5%	8.2%	35.7%	100%

Table S3. The evaluation of overlap count between two MβCD samples using both DEP f_{co} and V_{mbd}.

DEP f_{co} and V_{mbd}	**0 mM**	**2.5 mM**	**5 mM**	**10 mM**
**0 mM**	100%	-	-	-
**2.5 mM**	11.1 %	100%	-	-
**5.0 mM**	1.6%	20.2%	100%	**-**
**10 mM**	0%	1.5%	16.1%	100%

Table S4. Biological variability in bioelectrical measurement of SK-BR-3 cells.

**Biological replicate**	**1**	**2**	**3**
n (cells)	113	77	148
f_{co} (kHz)	8.24±2.36	8.18±3.58	7.56±2.53
V_{mbd} (V_{p-p})	5.56±0.76	5.89±0.71	6.11±0.82
Radius (μm)	8.81±0.99	8.71±0.939	8.83±0.98

Table S5. Biological variability in bioelectrical measurement of MDA-MB-231 cells.

**Biological replicate**	**1**	**2**	**3**
n (cells)	76	105	100
f_{co} (kHz)	3.86±1.64	3.43±1.18	3.54±1.46
V_{mbd} (V_{p-p})	8.84±1.05	8.97±1.50	9.18±1.52
Radius (μm)	8.73±0.91	8.63±1.3	8.87±1.56

4. Reference

1. Choi, S.; Lee, H.; Lee, S.; Park, I.; Kim, Y. S.; Key, J.; Lee, S. Y.; Yang, S.; Lee, S. W. A novel automatic segmentation and tracking method to measure cellular dielectrophoretic mobility from individual cell trajectories for high throughput assay. *Comput. Meth. Programs Biomed.* **2020**, 195, 105662.
2. Han, A.; Yang, L.; Frazier, A. B., Quantification of the Heterogeneity in Breast Cancer Cell Lines Using Whole-Cell Impedance Spectroscopy. *Clin. Cancer Res.* **2007**, 13 (1), 139-143.
3. Huang, C.; Liu, C.; Minne, B.; Ramirez Hernandez, J. E.; Stakenborg, T.; Lagae, L., Dielectrophoretic discrimination of cancer cells on a microchip. *Appl. Phys. Lett.* **2014,** 105 (14), 143702
4. Qiao, G.; Duan, W.; Chatwin, C.; Sinclair, A.; Wang, W., Electrical properties of breast cancer cells from impedance measurement of cell suspensions. *J. Exp. Clin. Cancer Res.* **2010**, 224 (1), 012081.
5. Park, I.; Lim, J. W.; Kim, S. H.; Choi, S.; Ko, K. H.; Son, M. G.; Chang, W.-J.; Yoon, Y. R.; Yang, S.; Key, J.; *et al.,* Bashir, R.; Lee, S. Y.; Lee, S. W., Variable Membrane Dielectric Polarization Characteristic in Individual Live Cells. *J. Phys. Chem. Lett.* **2020,** 11 (17), 7197-7203.
